# The Nursing Mirror

**Published:** 1888-01-28

**Authors:** 


### The Hospitals Association.

### The Nursing Mirror.

Communications for this column should be addressed The Mirror care of the Editor, 38, Old Jewry, London, E.C.

### ARE EXAMINATIONS DESIRABLE?

I have read with interest "M. B.'s" letter in this week's Hospital," and would like to make a few observations with regard to the topic therein raised.

In the face of the modern craze for examinations on every conceivable subject, a nurse with a certificate gained by examination undoubtedly possesses an advantages over her uncertificated sister. It is open to question, however, whether the art of nursing, an art in which practical so far outweighs theoretical knowledge, can be adequately tested by any examination.

Again, it should not be ignored that there are many excellent nurses whose ideas on the subject of examinations are such that nothing would induce them voluntarily to undergo one.

Apart from these objections, it seems to me that "M. B.'s" scheme would work in favour of well-to-do nurses as compared with poor ones, as very few of the latter would be able to spare the money for the travelling and other incidental expenses, which attendance at the proposed examination would entail.

In the meantime it would appear but just that every hospital that undertakes to train probationers, should grant a certificate at the expiry of the training should the nurse be held to deserve it; and I submit that it would be a very good thing could The Hospitals Association persuade them to do so.

January 21st, 1888. Kittifonia.

